# Functional Characterization of the *Almstn2* Gene and Its Association with Growth Traits in the Yellowfin Seabream *Acanthopagrus latus* (Hottuyn, 1782)

**DOI:** 10.3390/genes14122142

**Published:** 2023-11-27

**Authors:** Jianyi Guo, Huayang Guo, Chuanghua Chen, Fangzhao Yu, Baosuo Liu, Nan Zhang, Lin Xian, Zhiping Luo, Wen Liu, Kecheng Zhu, Dianchang Zhang

**Affiliations:** 1 Modern Agricultural Development Center of Zhuhai City, Zhuhai 519000, China; 13727030765@163.com (J.G.); cchwa@163.com (C.C.); yf200033@163.com (F.Y.); zhluozhp@163.com (Z.L.); zasliu202311@163.com (W.L.); 2 Key Laboratory of South China Sea Fishery Resources Exploitation and Utilization, Ministry of Agriculture and Rural Affairs, South China Sea Fisheries Research Institute, Chinese Academy of Fishery Sciences, 231 Xingang Road West, Guangzhou 510300, China; guohuayang198768@163.com (H.G.); liubaosuo343@163.com (B.L.); 398730316@163.com (N.Z.); c-xianlin@genomics.cn (L.X.); 3 Guangdong Provincial Engineer Technology Research Center of Marine Biological Seed Industry, Guangzhou 510300, China; 4 Sanya Tropical Fisheries Research Institute, Sanya 572018, China

**Keywords:** *Acanthopagrus latus*, mstn2, gene expression, SNP

## Abstract

Myostatin (mstn), also known as GDF8, is a growth and differentiation factor of the transforming growth factor-β (TGF-β) superfamily and plays a key inhibitory effect in the regulation of skeletal muscle development and growth in vertebrates. In the present study, to comprehend the role of the mstn2 gene of the yellowfin seabream *Acanthopagrus latus* (*Almstn2b*), the genomic sequence of *Almstn2b* is 2359 bp, which encodes 360 amino acids and is composed of three exons and two introns, was obtained. Two typical regions, a TGF-β propeptide and TGF-β domain, constitute *Almstn2b.* The topology indicated that *Almstn2* was grouped together with other Perciformes, such as the gilthead seabream *Sparus aurata*. Moreover, *Almstn2b* was mainly expressed in the brain, fins, and spleen. Furthermore, five SNPs, one in the exons and four in the introns, were identified in the *Almstn2b* gene. The allele and genotype frequencies of SNP-*Almstn2b* +1885 A/G were significantly related to the total weight, interorbital distance, stem length, tail length, caudal length, caudal height, body length, and total length (*p* < 0.05). The allele and genotype frequencies of SNP-*Almstn2b* +1888 A/G were significantly related to the weight, interorbital distance, long head behind the eyes, body height, tail length, caudal length, and body length. Additionally, the relationship between the SNP-*Almstn2b* +1915 A/G locus and weight and long head behind the eyes was significant (*p* < 0.05). Furthermore, the other two SNPs were not significantly associated with any traits. Thus, the SNPs identified in this study could be utilized as candidate SNPs for breeding and marker-assisted selection in *A. latus*.

## 1. Introduction

Yellowfin seabream, *A. latus*, a marine fish, belongs to Acanthopagrus, Sparidae, and Perciformes, and is widely distributed in the Red Sea, the Arabian Sea, the Indian Ocean, the western Pacific coast, and the coastal areas of Taiwan, Fujian, Guangdong, and Guangxi in China. It is an omnivorous fish and an important economic fish species in China [1,2]. In 2022, the annual output of Sparidae reached 130,000 tons [3]. However, the culture cycle of *A. latus* from fry to adulthood is long. It generally grows to market size after one to one and a half years of cultivation. In recent years, due to overfishing, environmental pollution, and other harmful conditions, the species quality and growth rate of the natural population of *A. latus* have seriously declined. Moreover, the species quality degradation of the cultured population is more serious, its immunity is decreased, and the profit of cultured *A. latus* is reduced. Therefore, it is urgent to implement working on culturing a new variety of *A. latus* [4]. Molecular-marker-assisted selection breeding is one of the commonly used methods for species breeding. Molecular markers closely linked to the target trait genes can be used for the association analysis of target traits, which can clarify the close association between candidate molecular markers and individual phenotypes, and further increase the rate of growth.

A single-nucleotide polymorphism (SNP) is a genetic marker that has many excellent characteristics. Firstly, there are a large number and wide distribution in the genome; secondly, some SNPs are located in the internal coding region of genes, and can be used as molecular selection sites for target traits; thirdly, the detection method is simple, and the large-scale experiment and detection automation requirements are low, which can greatly shorten the working time; and, lastly, an SNP is a single-nucleotide mutation with a low mutation rate, and has a higher genetic stability compared with repeat sequence markers such as microsatellites [2,5,6,7,8,9]. Therefore, they have been widely used in the research of growth traits related to aquatic animals.

Myostatin (mstn), also named growth and differentiation factor 8 (GDF8), is a GDF of the TGF-β superfamily that inhibits skeletal muscle growth and development [10]. In different species, the number of mstn paralog genes varies. There is only one mstn gene in mammalian genomes, and at least two in most ray-finned fish, with up to four in some species [11,12]. Compared to wild-type mice, mstn-knockout mice gained two to three times their muscle mass, and this is attributed to the hyperplasia and hypertrophy of the muscle fibers [13]. Starting with the fishery industry, inhibiting the expression of mstn is helpful for the management of muscle development in teleost fish, as well as for developing new policies to control the quality and production of meat. So far, numerous *mstn* genes have been identified in teleost fish, such as the gilthead seabream *S. aurata* [14], tilapia *Oreochromis mossambicus* [15], channel catfish *Ictalurus furcatus* [16], cyprinid loach *Misgurnus anguillicaudatus* [17], common carp *Cyprinus carpio* [18], zebrafish *Danio rerio* [19], rainbow trout *Oncorhynchus mykiss* [20], Japanese flounder *Paralichthys olivaceus* [21,22], yellowcheek carp *Elopichthys bambusa* [23], Atlantic salmon *Salmo salar* [24,25], mandarin fish *Siniperca chuatsi* [26], large yellow croaker *Larimichthys crocea* [27], grass carp *Ctenopharyngodon idellus* [28], Tibet fish *Gymnocypris przewalskii* [29], African lungfsh *Protopterus annectens* [30], pufferfish *Takifugu bimaculatus* [31], Nile tilapia *Oreochromis niloticus* [32], European sea bass *Dicentrarchus labrax* [33], and *A. latus* [34]. The functional identification of this gene lays the foundation for the genetic breeding of teleosts.

Additionally, many mammal studies have shown the presence of SNPs in the *mstn* gene [35,36]. In teleosts, nine SNPs and two indels were identified in exons 1–3 of mstn in the *D. labrax* population [33]. One of the SNPs which was scanned in the 5′-flanking region of the *mstn1b* gene had a prominent association with harvest traits in *S. salar* [25]. Moreover, *mstn* is a crucial candidate gene relevant to growth traits in *D. labrax* [37]. Although a lot of research has been conducted on mstn polymorphisms in teleost fish, this is lacking data useful for *A. latus*. In this study, the genomic DNA sequence of the *mstn2* gene in *A. latus* has been cloned. Three SNPs were identified and their association with the growth traits measured in a sample of *A. latus* was evaluated. The results of our study can provide available information for the selection and cultivation of *A. latus*.

## 2. Materials and Methods

### 2.1. Samples and Traits

Juvenile *A. latus* (the total length is 12.31 ± 1.20 cm and the body mass is 27.10 ± 7.30 g) were derived from Shenzhen Test Base, South China Sea Fisheries Research Institute, Chinese Academy of Fishery Sciences, Shenzhen, China. They were all full sibs reared under the same management and nutritional regimen. A total of 171 individuals were randomly selected to detect the SNPs. The body weight (BW), interorbital distance (ID), snout length (SL), eye diameter (ED), long head behind the eyes (LHBE), head length (HL), head height (HH), stem length (SL), body height (BH), tail length (TL), caudal length (CL), caudal height (CH), body length (BL), tail fin length (TFL), and body length (BL) of all individuals were measured. Moreover, adult fish tissues (n = 3), namely the brain, fins, spleen, small intestine, head kidney, skin, gills, heart, white muscle, liver, and stomach, were also collected.

### 2.2. DNA Extraction, PCR Amplification, and Sequencing

Based on the genomic sequences of *A. latus* [2], the Primer 5.0 software was applied to design multiple primers for the amplification of the *mstn* gene (Table 1). The genomic DNA in the muscle of *A. latus* was extracted using the traditional phenol/chloroform method. The extracted DNA solution was subjected to electrophoresis with 1% agarose gel, and the gel bands were analyzed to check the quality of the DNA extraction. Then, it was dissolved in sterile water at a concentration of 100 ng/mL and stored at −20 °C. Furthermore, PCR amplification was performed using a 100 μL reaction system, including 2× PCR MIX 50 μL, forward and reverse primers 2 μL each, DNA template 6 μL, and ddH_2_O 40 μL. Before the experiment began, temperature gradient PCR was performed to determine the optimal annealing temperature of the primers. The setting information of the PCR reaction was as follows: pre-denaturation at 94 °C for 4 min, 30 times; denaturing 40 s at 94 °C; annealing 40 s at optimum temperature; extending 90 s at 72 °C. After the end of the cycle, it was extended at 72 °C for 10 min and stored at 4 °C after extension. The DNA solution after PCR amplification was subjected to electrophoresis with 1% agarose gel, the gel bands were analyzed, and then the PCR products were sent to Beijing Tsingke Biotechnology Co., Ltd. for sequencing.

### 2.3. Bioinformatics of the Almstn2 Gene

The genome and amino acid sequences of the two mstn2 were searched for within the Ensembl (http://asia.ensembl.org/, accessed on 20 January 2023) and NCBI databases. Each mstn2s sequence was further verified using the NCBI database. According to the domain of known species, ClustalX2 was used to align and identify the domains of all mstn2s. The ORF finder (http://www.bioinformatics.org/sms2/orf_find.html, accessed on 20 January 2023) was used to recognize ORFs. Furthermore, MEGA 7.0 was used to structure the evolutionary relationship of mstn2 using the parameter of maximum likelihood (ML, bootstrap 1000) method [38].

### 2.4. Quantitative Real-Time PCR of the Almstn2 Gene

The tissue expression pattern of *Almstn2* was detected using quantitative real-time polymerase chain reaction (qRT-PCR) in *A. latus*. The tissue samples (spleen, fins, heart, kidney, small intestine, brain, gills, stomach, white muscle, skin, and liver) were obtained from six healthy adult fish. The specific primers for *Almstn2* and the housekeeping gene EF-1α (elongation factor 1, α) are listed in Table 1. The isolation of the total RNA and qRT-PCR was performed as previously described [5]. Relative expression was evaluated using the 2^−ΔΔCT^ method [39].

### 2.5. SNP Identification and Statistical Analysis

According to the data on body weight, PCR amplification and sequencing were performed on 10 DNA samples of bigger fish and 10 DNA samples of smaller fish, and ClustalX2 was used to compare the sequencing results to screen the loci with high polymorphism in the *mstn2* gene [40]. According to the selected SNP sites, Chromas was used to view the sequencing peak map of the location of the site, and then the site was classified. The genotype was determined using the peak map. When the peak height of the low peak in the peak map was less than half of the peak, the genotype of this locus was considered homozygous. When the peak height of the low peak of the bimodal reached or exceeded half of the peak, the genotype of this locus was considered to be heterozygous.

According to the genome sequence of *A. latus* (Table 1), short amplification primers containing SNP sites were designed, and 171 DNA samples of *A. latus* were amplified and sequenced for association analysis of various traits. After repeating the above steps, Excel tables were used to record the position of each SNP in the sample sequence and the corresponding genotype. Furthermore, to analyze the association between the gene polymorphism and traits, the R language’s aov and lm functions were used to fit an analysis of variance model and to fit a linear model, respectively. The linear analysis model was yij = u + Gi + eij. Here, yij was the trait phenotypic value, u was the population mean value, Gi was the marker genotype effect value, and eij was the random residual effect. The SPSS Statistics 17.0 software was used to conduct one-way ANOVA analysis on the SNP loci and various growth traits of *A. latus*, and to analyze whether there were significant differences between the SNP loci and various growth traits. Data are presented as the means of three replicates ± SE, and differences were considered significant at *p* < 0.05 and extremely significant at *p* < 0.01.

## 3. Results

### 3.1. Sequence Characterization of Almstn2b

**Figure 1:**
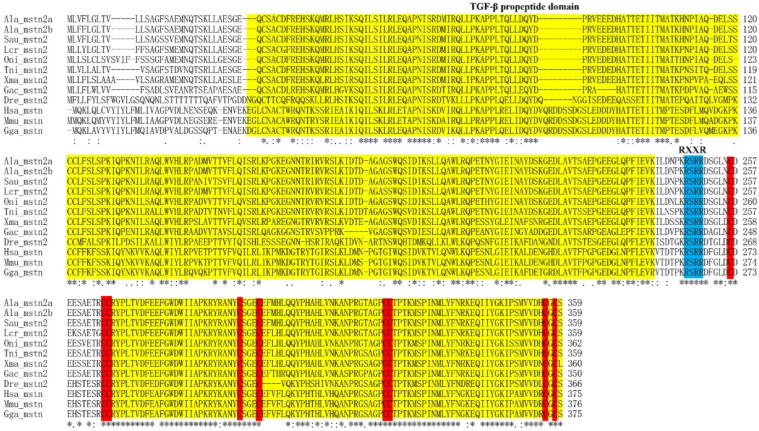
Amino acid sequences of mstn1 homologs in vertebrates. The TGF-β propeptide and TGF-β domains are indicated by yellow boxes; the conserved proteolytic cleavage site (RXXR) is indicated by a blue color box. The red boxes indicate the conserved cysteine residues present in all TGF-β family members. Identical (asterisks) and similar (: or .) residues identified using the Clustal X2 program are indicated. The Latin abbreviation and accession numbers are listed in Table S1.

The cDNA of *Almstn2b* is 2359 bp (GenBank accession number: OR494063), containing an open reading frame (ORF) of 1080 bp. The total protein of *Almstn2b* encodes 360 amino acid (aa) residues with a predicted molecular mass of 40.8 kDa and a theoretical pI of 6.33. The putative Almstn2b aa sequence was composed of two highly conserved domains: the TGF-β propeptide and TGF-β domains, which contained a conserved cysteine of a mature peptide, and a conserved RXXR proteolytic cleavage site (RSRR) (Figure 1).

Comparing the protein sequence of both Almstn2a and its paralog (Almstn2b), they displayed 99.8% similarity in identity. Additionally, Almstn2b exhibited a high degree of amino acid identity to gilthead seabream mstn2 (98.3%), spotted gar *Lepisosteus oculatus* mstn2 (96.4%), large yellow croaker mstn2 (96.4%), and Nile tilapia mstn2 (95.4%). However, there was a relatively low sequence identity similarity to higher vertebrates such as *Homo sapiens* (61.8%), *Mus musculus* (61.3%), and *Gallus gallus* (59.4%) (Table S1).

**Figure 2:**
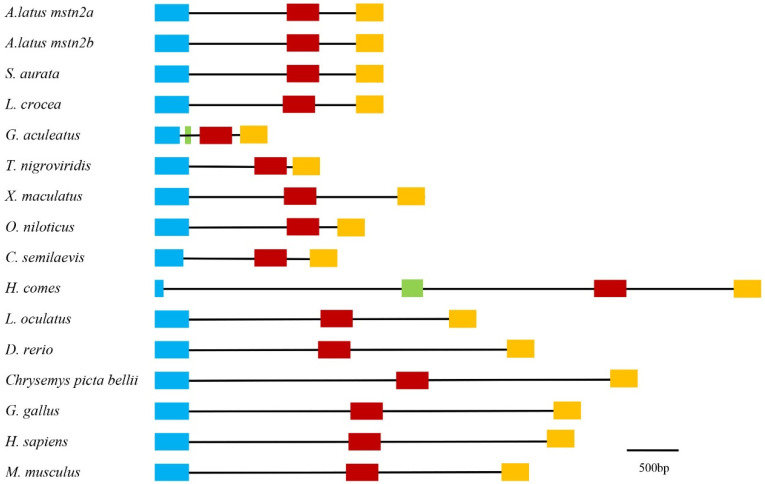
Genomic structure of *mstn2* gene. Lengths of exons and introns of each mstn2 gene are displayed proportionally. Different color boxes and lines represent exons and introns, respectively. The identical color boxes represent homologous sequences.

Three exons and two introns were identified in the *Almstn2* genes by comparing the ORF sequence with the reported genomic sequences in the Ensembl database. Interestingly, those species had three exons and two introns, except for the three-spined stickleback *Gasterosteus aculeatus* and seahorse *Hippocampus comes*, which possessed four exons and three introns. Actually, the first and second exons of stickleback and seahorse were derived from the first exon of other species (Figure 2, Table S2). Moreover, the lengths of the second and third exons were minorly different in the orthologous gene, suggesting that the sequences are comparatively conserved.

### 3.2. Phylogenetic Analysis

**Figure 3:**
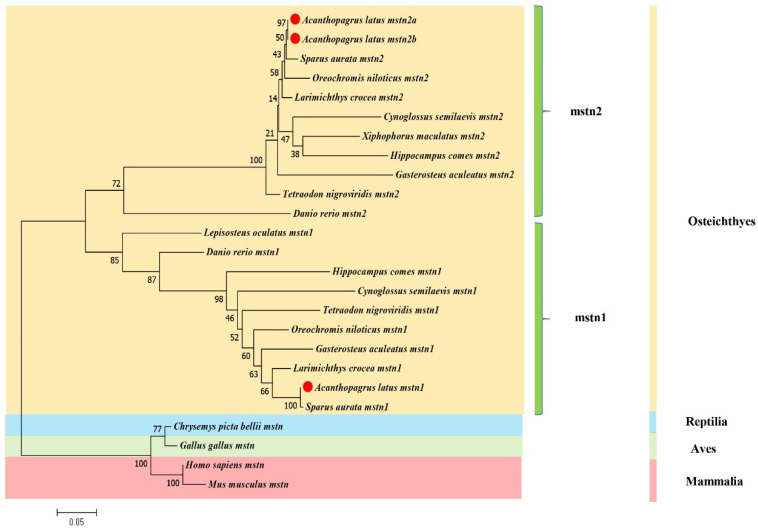
Evolutionary relationship of Almstn and other metazoan mstn genes. The tree depicts the overall sequences using the maximum likelihood (ML) method of the MEGA 7.0 software. Pink, green, blue, and yellow shows Mammalia, Aves, Reptilia, and Osteichthyes, respectively. The accession numbers of the mstn sequences are listed in Table S1. The bar stands for the scale length and the numbers on different nodes stand for bootstrap values. The identical color boxes represent homologous sequences.

We downloaded 25 sequences of the mstn gene from 15 species, including *A. latus*, *S. aurata*, *L. crocea*, *O. niloticus*, the tongue sole Cynoglossus semilaevis, *G. aculeatus*, the platyfish *Xiphophorus maculatus*, the green-spotted puffer fish *Tetraodon nigroviridis*, *H. comes*, *L. oculatus*, *D. rerio*, the Western painted turtle *Chrysemys picta bellii*, *G. gallus*, *H. sapiens*, and *M. musculus* (Table S3). To structure the phylogenetic tree, the protein sequences of these species were aligned using Clustal W2 and evolutionary relationships were established using MEGA 7.0. with the ML method. The sequences mainly clustered into three main clades: one and two were mstn1 and mstn2 clusters containing only teleosts, respectively, and the other was an mstn2 cluster containing Reptilia, Aves, and Mammalia (Figure 3). Additionally, Almstn1 and Almstn2 are most closely related to the evolution of mstn1 and mstn2 in *S. aurata*, respectively, suggesting that the sequences between *A. latus* and *S. aurata* are highly homologous.

### 3.3. Tissue Expression of Almstn2b

**Figure 4:**
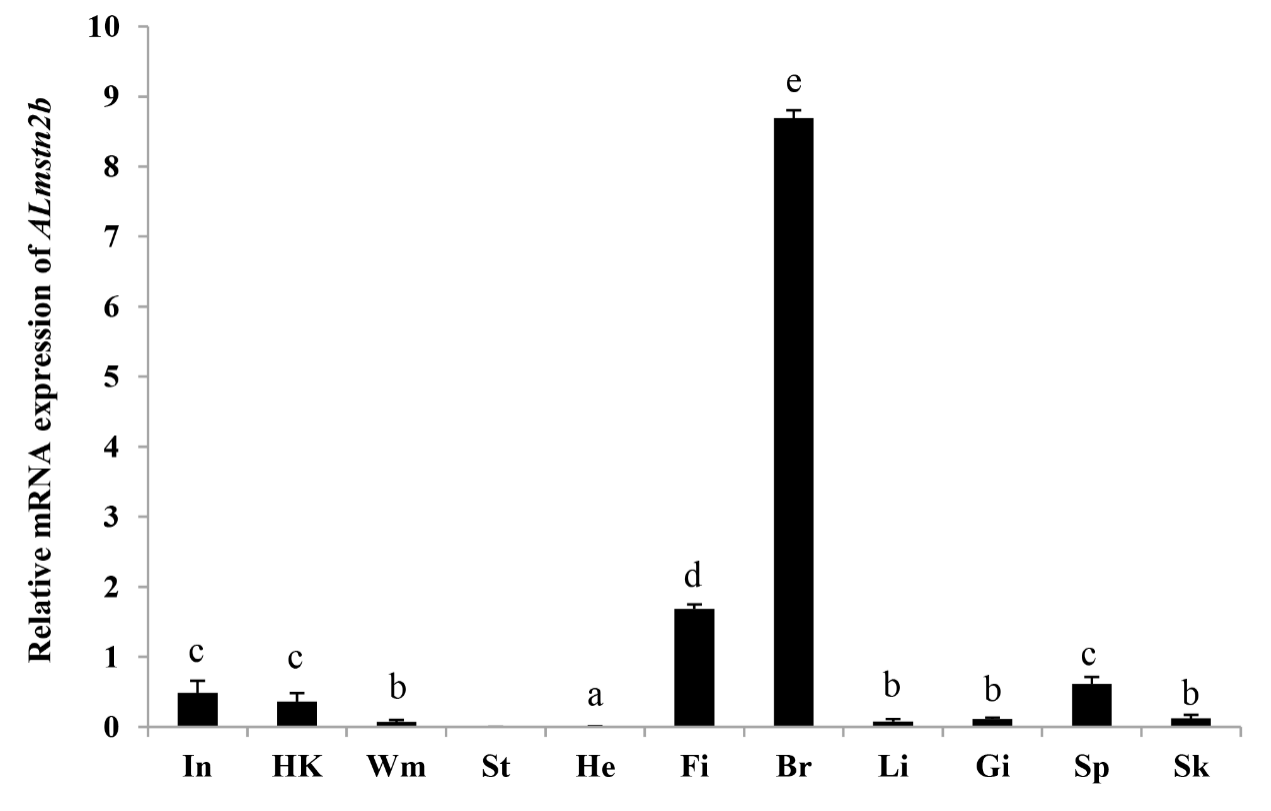
Gene transcriptions of *Almstn2b* in various tissues. The 11 tissues are spleen (Sp), fins (Fi), heart (He), kidney (Ki), small intestine (In), brain (Br), gills (Gi), stomach (St), white muscle (Wm), skin (Sk), and liver (Li). Different letters indicate significant differences.

The mRNA expression levels of mstn2b were detected using qRT-PCR in 11 tissues of *A. latus*. The transcriptions of *Almstn2* were high in the brain and fins, moderate in the spleen, small intestine, and head kidney, and low in the other tissues (Figure 4). Additionally, this gene expression was absent in the stomach.

### 3.4. Polymorphism of the Almstn2b Gene

The genome DNA of 10 individuals was randomly selected. The genomic sequence of *mstn2b* was obtained via the amplification of specific primers (Table 1). Mixed-pool sequencing revealed that there was one SNP in the exon of the *mstn2b* gene, which was named SNP-*Almstn2b* +1700 C/A, and four SNPs in the intron, which were named SNP-*Almstn2b* +1885 A/G, SNP-*Almstn2b* +1888 A/G, *Almstn2b* +1901 C/T, and SNP-*Almstn2b* +1915 A/G, respectively.

### 3.5. Associations between the Genotypes of mstn2b and Growth Traits

After linear model analysis, the correlation analysis results of the different genotypes of SNP-*Almstn2b* +1885 A/G (Table 2), SNP-*Almstn2b* +1888 A/G (Table 3), SNP-*Almstn2b* +1915 A/G (Table 4), and the growth traits were completed. However, SNP-*Almstn2b* +1700 C/A and *Almstn2b* +1901 C/T were not significantly associated with any traits.

From Table 2, the body weight of individuals with the AA genotype at the SNP-*Almstn2b* +1885 A/G site was significantly greater than that of AG or GG genotype individuals (*p* < 0.01). The interorbital distance of AA genotype individuals was dramatically higher than that of AG or GG genotype individuals (*p* < 0.01). The stem length of AA genotype individuals was larger than that of AG genotype individuals (*p* < 0.05). Moreover, the tail of individuals with the AA genotype at the SNP locus was larger than that of AG or GG genotype individuals (*p* < 0.01). The tail length, caudal length, and body length of AA genotype individuals were observably larger than those of AG or GG genotype individuals, respectively (*p* < 0.01). The caudal height and total length of individuals with the AA genotype at the SNP locus were greater than those of AG or GG genotype individuals, respectively (*p* < 0.05).

From Table 3, the body weight and interorbital distance of individuals with the GG genotype at the SNP-*Almstn2b* +1888 A/G site was significantly greater than that of AG or AA genotype individuals, respectively (*p* < 0.01). Furthermore, the long head behind the eyes, tail length, caudal length, and body length of individuals with the GG genotype at the SNP-*Almstn2b* +1888 A/G site was significantly larger than that of AG or AA genotype individuals, respectively (*p* < 0.05). In addition, the body height of individuals with the GG genotype at the SNP locus was greater than that of AG genotype individuals (*p* < 0.05), and there was no significant difference between GG genotype and AA genotype individuals (*p >* 0.05).

As shown in Table 4, the body weight and long head behind the eyes of individuals with the GG genotype at the SNP-*Almstn2b* +1915 A/G site were significantly greater than those of AG or AA genotype individuals, respectively (*p* < 0.05).

## 4. Discussion

mstn plays an inhibitory role in skeletal muscle growth and development. In aquatic animals, it is mainly a key growth-related candidate gene. In the LG15 of *D. labrax*, mstn and insulin growth-factor-binding proteins (IGFBPs) are located in a prominent QTL (quantitative trait locus) interval for morphology [37]. Likewise, according to genome-wide association studies (GWAS), mstn1 is one of the growth-related genes in *T. bimaculatus* [41]. Moreover, as a key breeding candidate gene in *L. crocea*, mstn was implicated in the regulation of morphology determination [42]. Furthermore, in the analysis of SNP loci and morphology trait association, the total weight, fillet weight, and standard length was significantly related to the mstn SNP5252T > A locus in intron 2 in *D. labrax* [33]. The relationship between the abdominal length, pre-anal length, post-anal length, standard height, head length, head width, and body length and SNP14873C > T locus in exon 3 of *D. labrax* mstn was prominent (*p* < 0.05) [33]. In *S. salar*, three original SNPs were acquired in the *mstn1b* gene. SNP1086C > T and the weight traits (HWT, GWT, DHWT, and FLWT) were significantly associated, which showed a high positive correlation (r > 0.97) [26]. In the present study, we used sequencing to detect three SNPs (SNP1885, SNP1888, and SNP1915) in the mstn intron in the *A. latus* population. All three loci of SNP-*Almstn2b* +1885 A/G, SNP-*Almstn2b* +1888 A/G, and SNP-*Almstn2b* +1915 A/G had a significant association with the morphological characteristics, especially weight traits, which were similar to the above study [26,33].

On account of the low selection pressure for the promoter and intronic regions, the polymorphism sites in those two regions were more frequent than in the exonic regions [43,44]. Regardless, in non-coding regions, SNPs were involved in influencing phenotypic variation and regulating gene expression [45,46,47]. For instance, in *O. mykiss*, the SNP1904C > A locus is associated with body length and body weight in intron 2 of *mstn1* [48]. Similarly, all three SNP loci, which had significant associations with growth traits, were in the intronic regions in *A. latus*. However, this does not mean that these sites are not important. Further experiments are needed for analysis. In mammals, the deletion of 11 base pairs on the third exon of *mstn* could improve muscle mass by 20–25% compared to the control group via hyperplasia in Belgian blue cattle [49,50]. In fish, no genetic variants with distinct phenotypes have been identified [51]. Our three SNPs should be further evaluated in terms of their effect in other populations of *A. latus*. Therefore, future studies should aim at evaluating the effect of additional mstn polymorphisms and other candidate genes for growth in *A. latus*.

In mammals and birds, one paralog gene *mstn* was primarily transcripted in the skeletal muscle, brain, and heart [21,27,52,53]. Nevertheless, the mstn gene was ubiquitous in various organs in teleost fish [22,32]. Multiple paralogous mstn genes result in different expression levels in different species [15,54]. In *D. rerio*, mstn1 and mstn2 had prominent differences in function, expression pattern, and structure [15]. Moreover, mstn was expressed in the brain in all known teleost fish, especially *mstn2*, which was generally expressed at a high level in the brain. In the present study, the expression results are similar to those of previous studies. The mstn2b expressions were ubiquitous except for in the stomach, and were highest in the brain, suggesting that mstn2 played an important role in the brain and might be involved in the development of the nervous system in teleost fish.

In this study, the Almstn2b protein was composed of 360 amino acids, and Almstn2b shared a high identity with Almstn2a (99.8%). In rainbow trout, mstn2a and mstn2b also revealed a correspondingly high aa sequence identity of 81% [20], suggesting that these paralogs were relatively conserved. Moreover, in teleosts, phylogenetic analysis showed that mstn1 and mstn2 were grouped into two clusters, which was completed in accordance with the previous study [20,29,31].

## 5. Conclusions

In conclusion, the function of the *mstn2* gene was analyzed in this study. Furthermore, the three SNP sites, SNP-*Almstn2b* +1885 A/G, SNP-*Almstn2b* +1888 A/G, and SNP-*Almstn2b* +1915 A/G, were all dramatically related to the body weight traits of *A. latus*. These three SNP sites could be applied to the early screening of breeding materials of *A. latus* aiming at rapid growth, which can effectively improve the breeding efficiency and shorten the culture cycle.

## Figures and Tables

**Table 1:** Primers used for sequence cloning.

Subject and Primers	Nucleotide Sequence	Tm (°C)
M2b-G-R1	GAGCACAGCAAGCAGATG	56
M2b-G-L1	CTTTGGAGTCGTAGGCGT	
M2b-G-R2	AACAACACCCGAATCAGA	56
M2b-G-L2	CCAGGGACGAGAAGACC	
M2b-SNP-R2	TTCTTCTTCCAGACAATCC	56
M2b-SNP-L2	AAAAAGTGCCACATCCC	

**Table 2:** Association analysis of different genotypes and growth traits at SNP1885 locus from *mstn2a*.

Traits	Genotype			
	AA	AG	GG	X^2^ (*p*)
BW/g	34.56 ± 6.41 ^a^	25.51 ± 7.97 ^b^	27.31 ± 6.64 ^b^	0.001
ID/cm	1.45 ± 0.08 ^a^	1.32 ± 0.12 ^b^	1.36 ± 0.10 ^b^	0.002
SL/cm	0.65 ± 0.23	0.65 ± 0.13	0.69 ± 0.20	0.243
ED/cm	0.99 ± 0.10	0.98 ± 0.11	1.00 ± 0.11	0.555
LHBE/cm	1.50 ± 0.10	1.40 ± 0.17	1.37 ± 0.17	0.089
HL/cm	3.14 ± 0.34	3.02 ± 0.33	3.07 ± 0.37	0.552
HH/cm	3.95 ± 0.31	3.74 ± 0.42	3.83 ± 0.36	0.173
SL/cm	4.63 ± 0.44 ^a^	4.17 ± 0.63 ^b^	4.33 ± 0.50 ^ab^	0.029
BH/cm	4.43 ± 0.28	4.14 ± 0.49	4.25 ± 0.41	0.087
TL/cm	3.05 ± 0.30 ^a^	2.66 ± 0.39 ^b^	2.74 ± 0.36 ^b^	0.009
CL/cm	2.21 ± 0.18 ^a^	1.93 ± 0.30 ^b^	1.99 ± 0.24 ^b^	0.008
CH/cm	1.47 ± 0.13 ^a^	1.32 ± 0.24 ^b^	1.37 ± 0.14 ^ab^	0.046
BL/cm	10.82 ± 0.68 ^a^	9.85 ± 1.06 ^b^	10.14 ± 0.90 ^b^	0.009
TFL/cm	2.37 ± 0.19	2.22 ± 0.36	2.21 ± 0.38	0.408
TL/cm	13.19 ± 0.81 ^a^	12.07 ± 1.29 ^b^	12.35 ± 1.15 ^b^	0.021

Note: BW body weight, ID interorbital distance, SL snout length, ED eye diameter, LHBE long head behind the eyes, HL head length, HH head height, SL stem length, BH body height, TL tail length, CL caudal length, CH caudal height, BL body length, TFL tail fin length, BL body length. Different letters indicate the significance of the difference, *p* < 0.05 indicates significant difference, *p* < 0.01 indicates extremely significant difference.

**Table 3:** Association analysis of different genotypes and growth traits at SNP1888 locus from *mstn2a*.

Traits	Genotype			
	AA	AG	GG	X^2^ (*p*)
BW/g	27.26 ± 6.7 ^b^	25.38 ± 7.73 ^b^	33.87 ± 6.92 ^a^	0.001
ID/cm	1.36 ± 0.10 ^b^	1.32 ± 0.12 ^b^	1.43 ± 0.08 ^a^	0.002
SL/cm	0.69 ± 0.20	0.65 ± 0.14	0.67 ± 0.21	0.368
ED/cm	1.00 ± 0.11	0.97 ± 0.11	1.01 ± 0.09	0.235
LHBE/cm	1.37 ± 0.18 ^b^	1.39 ± 0.17 ^b^	1.51 ± 0.10 ^a^	0.022
HL/cm	3.07 ± 0.37	3.01 ± 0.33	3.19 ± 0.31	0.227
HH/cm	3.83 ± 0.36	3.73 ± 0.41	3.99 ± 0.29	0.051
SL/cm	4.33 ± 0.51	4.18 ± 0.63	4.52 ± 0.46	0.082
BH/cm	4.25 ± 0.41 ^ab^	4.13 ± 0.49 ^b^	4.47 ± 0.29 ^a^	0.029
TL/cm	2.74 ± 0.36 ^b^	2.67 ± 0.39 ^b^	2.97 ± 0.34 ^a^	0.034
CL/cm	1.99 ± 0.24 ^b^	1.94 ± 0.30 ^b^	2.13 ± 0.22 ^a^	0.049
CH/cm	1.37 ± 0.14	1.32 ± 0.24	1.44 ± 0.14	0.076
BL/cm	10.13 ± 0.91 ^b^	9.86 ± 1.06 ^b^	10.69 ± 0.73 ^a^	0.015
TFL/cm	2.20 ± 0.38	2.24 ± 0.34	2.29 ± 0.34	0.647
TL/cm	12.33 ± 1.17	12.1 ± 1.29	12.97 ± 0.94	0.060

Note: BW body weight, ID interorbital distance, SL snout length, ED eye diameter, LHBE long head behind the eyes, HL head length, HH head height, SL stem length, BH body height, TL tail length, CL caudal length, CH caudal height, BL body length, TFL tail fin length, BL body length. Different letters indicate the significance of the difference, *p* < 0.05 indicates significant difference, *p* < 0.01 indicates extremely significant difference.

**Table 4:** Association analysis of different genotypes and growth traits at SNP1915 locus from *mstn2a*.

Traits	Genotype			
	AA	AG	GG	X^2^ (*p*)
BW/g	27.19 ± 6.72 ^b^	25.55 ± 7.99 ^b^	31.19 ± 7.71 ^a^	0.017
ID/cm	1.36 ± 0.10	1.32 ± 0.12	1.39 ± 0.10	0.053
SL/cm	0.69 ± 0.20	0.64 ± 0.14	0.66 ± 0.19	0.279
ED/cm	1.00 ± 0.12	0.97 ± 0.11	1.01 ± 0.11	0.236
LHBE/cm	1.38 ± 0.18 ^b^	1.38 ± 0.17 ^b^	1.49 ± 0.12 ^a^	0.025
HL/cm	3.07 ± 0.37	2.99 ± 0.34	3.17 ± 0.30	0.164
HH/cm	3.83 ± 0.37	3.72 ± 0.41	3.93 ± 0.33	0.077
SL/cm	4.32 ± 0.50	4.21 ± 0.65	4.37 ± 0.55	0.458
BH/cm	4.25 ± 0.42	4.13 ± 0.49	4.35 ± 0.38	0.133
TL/cm	2.75 ± 0.37	2.68 ± 0.39	2.80 ± 0.41	0.403
CL/cm	1.99 ± 0.25	1.94 ± 0.30	2.06 ± 0.26	0.185
CH/cm	1.37 ± 0.14	1.32 ± 0.26	1.40 ± 0.14	0.215
BL/cm	10.13 ± 0.91	9.89 ± 1.08	10.34 ± 0.95	0.157
TFL/cm	2.20 ± 0.38	2.23 ± 0.33	2.28 ± 0.37	0.667
TL/cm	12.34 ± 1.16	12.12 ± 1.31	12.62 ± 1.14	0.273

Note: BW body weight, ID interorbital distance, SL snout length, ED eye diameter, LHBE long head behind the eyes, HL head length, HH head height, SL stem length, BH body height, TL tail length, CL caudal length, CH caudal height, BL body length, TFL tail fin length, BL body length. Different letters indicate the significance of the difference, *p* < 0.05 indicates significant difference, *p* < 0.01 indicates extremely significant difference.

## Data Availability

Data is contained within the article or supplementary material.

## Supplementary Materials

The following supporting information can be downloaded at: https://www.mdpi.com/article/10.3390/genes14122142/s1, Table S1: mstn2a proteins used in multiple alignment; Table S2: Lengths of exons (bp) and introns (bp) of each mstn2 gene; Table S3: Mstn1 and mstn2 proteins used in this study.
