# Supplementary material for: Functional Characterization of the Almstn2 Gene and Its Association with Growth Traits in the Yellowfin Seabream Acanthopagrus latus (Hottuyn, 1782)
Source: Genes (Basel). 2023 Nov 27;14(12):2142. doi: 10.3390/genes14122142 (PMC10742913; doi:10.3390/genes14122142)
Supplement: Supplementary file 1 [file genes-14-02142-s001.zip › genes-2720298-supplementary.pdf]

Supplementary Table

Table S1. mstn2a proteins used in multiple alignment.

Species	Protein name	Total AA identity %
Acanthopagrus latus	mstn2b	100
Acanthopagrus latus	mstn2a	99.8
Sparus aurata	mstn2	98.3
Lepisosteus oculatus	mstn2	96.4
Larimichthys crocea	mstn2	96.4
Oreochromis niloticus	mstn2	95.4
Tetraodon nigroviridis	mstn2	93.6
Xiphophorus maculatus	mstn2	86.6
Gasterosteus aculeatus	mstn2	82.2
Hippocampus comes	mstn2	81.0
Cynoglossus semilaevis	mstn2	80.5
Chrysemys picta bellii	mstn	80.5
Danio rerio	mstn2	62.5
Homo sapiens	mstn2	61.8
Mus musculus	mstn2	61.3
Gallus gallus	mstn2	59.4

Table S2. Lengths of exons (bp) and introns (bp) of each mstn2 gene.

Species	E1	I1	E2	I2	E3	I3	E4
Acanthopagrus latus mstn2a	328	969	371	323	381	-	-
Acanthopagrus latus mstn2b	328	956	371	323	381	-	-
Sparus aurata	328	940	371	341	381	-	-
Larimichthys crocea	328	877	371	435	381	-	-
Gasterosteus aculeatus	271	21	42	195	359	120	381
Tetraodon nigroviridis	325	642	374	69	381	-	-
Xiphophorus maculatus	331	829	371	932	381	-	-
Oreochromis niloticus	340	849	368	264	381	-	-
Cynoglossus semilaevis	280	693	371	253	381	-	-
Hippocampus comes	76	4659	243	2003	365	1594	381
Lepisosteus oculatus	373	1209	1074	381	371	-	-
Danio rerio	361	1202	2201	369	371	-	-
Chrysemys picta bellii	373	2656	3133	381	374	-	-
Gallus gallus	373	2091	2274	381	374	-	-
Homo sapiens	374	1788	2423	381	374	-	-
Mus musculus	376	1741	1994	381	374	-	-

Table S3. Mstn1 and mstn2 proteins used in this study.

Species	Abbreviation	Protein name	No.	Protein name	No.
Acanthopagrus latus	Ala	mstn1	OR735568	mstn2a<br>mstn2b	OR735569<br>OR735570
Sparus aurata	Sau	mstn1	ENSSAUT00010010370.1	mstn2	ENSSAUT00010053325.1
Larimichthys crocea	Lcr	mstn1	ENSLCRT00005045592.1	mstn2	ENSLCRT00005044672.1
Oreochromis niloticus	Oni	mstn1	ENSONIT00000048569.1	mstn2	ENSONIT00000015370.2
Cynoglossus semilaevis	Cse	mstn1	XP_008327219.1	mstn2	ENSCSET00000008642.1
Gasterosteus aculeatus	Gac	mstn1	ENSSAUT00010010370.1	mstn2	ENSGACT00000020597.1
Xiphophorus maculatus	Xma	mstn1	——	mstn2	ENSXMAT00000021209.1
Tetraodon nigroviridis	Tni	mstn1	ENSTNIT00000019017.1	mstn2	ENSTNIT00000020527.1
Hippocampus comes	Hco	mstn1	ENSHCOT00000004812.1	mstn2	ENSHCOT00000001635.1
Lepisosteus oculatus	Loc	mstn1	ENSLOCT00000010751.1	mstn2	——
Danio rerio	Dre	mstn1	ENSDART00000100386.2	mstn2	ENSDART00000063055.5
Chrysemys picta bellii	Xtr	mstn	ENSCPBT00000034185.1	mstn2	——
Gallus gallus	Gga	mstn	ENSGALT00010030820.1	mstn2	——
Homo sapiens	Hsa	mstn	ENST00000260950.5	mstn2	——
Mus musculus	Msu	mstn	ENSMUST00000027269.7	mstn2	——
